# Golgi Phosphoprotein 3 Represents a Novel Tumor Marker for Gastric and Colorectal Cancers

**DOI:** 10.1155/2021/8880282

**Published:** 2021-02-23

**Authors:** Chun-Xiao Wang, Hai-Bin Zhuang, Ze-Sheng Shi, Cheng-Zhi Qiu, Zhi-Xiong Chen, Long-Feng Tang

**Affiliations:** ^1^Department of General Surgery, The Second Affiliated Hospital of Fujian Medical University, Quanzhou, Fujian Province, China; ^2^Department of Gastrointestinal Surgery, Quanzhou First Hospital Affiliated to Fujian Medical University, China; ^3^Department of General Surgery, Xiamen Hospital of T.C.M., Xiamen, Fujian Province, China; ^4^Department of Orthopedics, ANXI County Hospital, Quanzhou, Fujian Province, China

## Abstract

**Background:**

Early diagnosis is very important for the clinical treatment of gastric cancer (GC) and colorectal cancer (CRC). We aimed to detect Golgi phosphoprotein 3 (GOLPH3) and evaluate its diagnostic value.

**Materials and Methods:**

Serum concentrations of GOLPH3 were detected by ELISA in 136 CRC patients, 102 GC patients, and 50 healthy controls at the Second Affiliated Hospital of Fujian Medical University from June 2016 to December 2019. Serum concentrations of CEA and CA19-9 were detected by ECLIA.

**Results:**

Serum concentrations of GOLPH3, CEA, and CA19-9 were higher in GC and CRC patients than in healthy controls (*P* < 0.001). Serum GOLPH3 concentrations were increased in GC and CRC patients with tumors greater than 5 cm, poor differentiation, greater depth of tumor invasion, and increased lymphatic and distant metastases (*P* < 0.05). In the GC and CRC groups, the AUCs of GOLPH3 were higher than those of CEA and CA19-9 (*P* < 0.05), while the AUCs of the marker combination were higher than those of GOLPH3 (*P* < 0.05), and postoperative serum GOLPH3 levels were lower than preoperative levels (*P* < 0.001). Serum GOLPH3 concentrations in CRC patients correlated positively with CEA and CA19-9 concentrations (*P* < 0.05).

**Conclusion:**

Serum GOLPH3 concentrations in GC and CRC patients are related to TNM stage. GOLPH3 may represent a novel biomarker for the diagnosis of GC and CRC. The combination of serum GOLPH3, CEA, and CA19-9 concentrations can improve diagnostic efficiency for GC and CRC. GOLPH3 is expected to become an indicator for the early diagnosis and evaluation of surgical effects.

## 1. Introduction

According to the 2018 GLOBOCAN report published by the International Agency for Research on Cancer (IARC), gastric and colorectal cancers (GC and CRC, respectively) account for 15.9% of new cancer cases and 17.4% of the total number of deaths, higher than for other cancers [1]. Screening of healthy people can allow for early diagnosis of tumors, thereby effectively reducing the incidence and mortality of GC and CRC. Serum tumor markers have definite value for the early diagnosis of tumors, but the sensitivity and specificity of serum carcinoembryonic antigen (CEA) and antigen199 (CA19-9) are limited, which are commonly used for the diagnosis of GC and CRC. Therefore, the search for new serum tumor markers is of great significance.

Recent studies have indicated that Golgi phosphoprotein 3 (GOLPH3) acts as a peripheral membrane protein in the trans-Golgi network (TGN). GOLPH3 is a novel oncoprotein that correlates with cell signal transduction and is potentially mobilized by stress [2]. In a study by Hu et al., serum GOLPH3 concentrations in GC patients were significantly higher than those in healthy people, though the clinical value of serum GOLPH3 for GC was not further evaluated [3]. In addition, Fan et al. reported that the serum GOLPH3 level in ovarian cancer patients was significantly higher than that in healthy people. Moreover, the serum GOLPH3 level in patients was significantly lower after than before surgery. It has been suggested that the serum GOLPH3 concentration can be considered an index for the diagnosis of ovarian cancer and evaluation of surgical effects [4]. By analyzing the difference in GOLPH3 expression between cancer patients and healthy individuals, Lu et al. found that serum GOLPH3 has early diagnostic value for bladder cancer, with an area under the ROC curve (AUC) of 0.948 and specificity and sensitivity of 92.5% and 83.8%, respectively [5]. The clinical value of the serum GOLPH3 concentration in other cancers has not been reported.

This study is aimed at detecting the serum GOLPH3 concentrations in GC and CRC patients and exploring its clinical significance.

## 2. Materials and Methods

### 2.1. Clinical Samples and Pathological Data

In total, 238 GC and CRC patients who were first diagnosed at the Second Affiliated Hospital of Fujian Medical University from June 2016 to December 2019, including 136 CRC patients and 102 GC patients, were enrolled; 50 healthy controls during the same period were also included. Serum samples were collected from all patients within 3 days before treatment; samples were collected from 225 patients who underwent radical surgery (128 CRC patients and 97 GC patients) 7 ± 2 days and 30 ± 2 days after surgery. All patients signed the trial ethics informed consent form, which was reviewed and approved by the hospital ethics committee. The eighth edition of the UICC/AJCC TNM staging system was used for GC and CRC clinical staging.

### 2.2. Oncomine Database Analysis

Before the start of this study, we used the Oncomine database (https://www.oncomine.org) to download an existing cancer microarray data set to clarify the mRNA expression status of GOLPH3 in GC and CRC. The Oncomine filter index was set with the search mode set to gene “GOLPH3”. The main filter settings were difference analysis “cancer and normal tissues”and cancer types “stomach cancer” and “colorectal cancer.” The data set filter was set to the data type “mRNA,” and the statistical results show the differential mRNA expression of GOLPH3 between GC/CRC and normal tissues, including sample size, *P* value, fold change, and statistical box plot.

### 2.3. Detection of Serum GOLPH3, CEA, and CA19-9 Concentrations

Fasting peripheral venous blood was collected (4-5 mL) from all patients and healthy controls. All samples were placed at room temperature for 2 h and centrifuged at 3000 r/min for 5 minutes. The sample was pipetted into a polypropylene tube (EP tube) and stored at -20°C. Samples in which hemolysis occurred were discarded. The samples were thawed at room temperature and mixed avoiding the production of bubbles.

Serum GOLPH3 concentrations were detected using an enzyme-linked immunosorbent assay kit (ELISA, purchased from MyBioSource) (standard curve range: 0.2 ng/mL-60 ng/mL; sensitivity: 0.11 ng/mL; intra-assay: CV < 8%; interassay: CV < 10%). The plate well strips were equilibrated at room temperature for 30 minutes and divided into standard wells and sample wells. Fifty microliters of the standard solution was pipetted, and 20 *μ*L of the sample and 30 *μ*L of the dilution solution (diluted 2.5 times) were simultaneously added to the plate. In addition to blank wells, 100 *μ*L of horseradish peroxidase- (HRP-) labeled detection antibody was added to each of the standard wells and the sample wells, and the plates were incubated at 37°C for 60 minutes. The plates were then washed; 350 *μ*L of washing solution was diluted and used to wash the samples 4 times in total. Next, 50 *μ*L of each of the chromogenic solutions A and B was added to each well. After incubating at 37°C in the dark for 15 minutes, 50 *μ*L of the reaction stop solution was added to each well to terminate the reaction. The OD450 value was assessed within 15 minutes. The absorbance value of the standard solution was calculated, taking the logarithm of the standard solution concentrations as the *X* axis and the logarithm of the absorbance of the standard solution as the *Y* axis; a standard curve was drawn using an Excel worksheet. The corresponding sample concentrations were determined on the curve using the light absorption value for different samples. The result was multiplied by a dilution factor of 2.5 to obtain the final sample concentrations in ng/mL.

Using electrochemiluminescence immunoassay (ECLIA), a CEA quantitative determination kit and CA19-9 determination kit were purchased from Roche Diagnostics Co., Ltd., to detect serum CEA and CA19-9 concentrations. The normal reference value range for CEA was assumed to be 0-5.2 ng/mL and that for CA19-9 to be 0-37 U/mL. The experimental operations were performed strictly in accordance with the instructions.

### 2.4. Statistical Analysis

Statistical analysis was performed using IBM SPSS statistics software, version 25.0 (IBM Corp., Armonk, NY, USA). The data of each group were firstly treated with a normal distribution test (Shapiro-Wilk test). In nonnormal distribution of measurement data, comparison between two groups was performed by using a Mann-Whitney *U* test, and differences between groups were compared by using a Kruskal-Wallis *H* test. The Wilcoxon test was employed to compare concentrations of serum GOLPH3 before and after surgery. The predictive ability of GOLPH3, CEA, CA19-9, and the combination of all 3 markers for the two cancers was determined by logistic regression. The receiver operating characteristic (ROC) curve was drawn in SPSS to calculate the area under the ROC curve (AUC) and 95% confidence interval (CI) of each tumor marker. The Youden index (sensitivity+specificity-1) was calculated with Excel. Comparisons of two AUCs were conducted with DeLong's algorithm by using MedCalc Statistical Software version 15.2.2 (MedCalc Software bvba, Ostend, Belgium). Spearman's correlation coefficient was calculated to assess correlation among GOLPH3, CEA, and CA19-9 expression levels. The statistical tests used above were two-sided, and a *P* value < 0.05 was considered significant. *P* values were adjusted by Bonferroni correction for multiple comparisons among groups. The corresponding experimental figures were drawn using GraphPad Prism version 8.3 software (GraphPad Software, Inc., La Jolla, CA, USA).

## 3. Results

### 3.1. Oncomine Database Analysis Confirms Upregulation of GOLPH3 mRNA in GC and CRC

**Figure 1:**
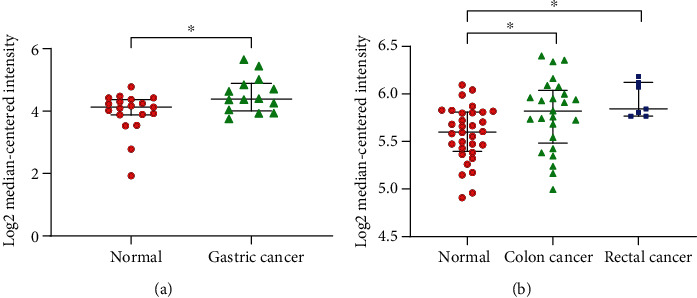
Scatter plots based on data downloaded from the Oncomine database show upregulation of GOLPH3 mRNA in GC and CRC patients relative to normal tissues: (a) Cho's data show the GOLPH3 mRNA in 14 GCs and 19 normal tissues; (b) Sabates' data show 32 normal tissues, 25 colon cancer patients, and 7 rectal cancer patients. ^∗^*P* < 0.05.

Based on a previous study showing that GOLPH3 protein expression is upregulated in GC and CRC tissues, we conducted data mining and analyzed the GOLPH3 transcription profile data set of existing data and compared mRNA expression of GOLPH3 in GC/CRC and normal tissues. According to statistical analysis of data downloaded from the publicly available Oncomine database, mRNA expression of GOLPH3 in GC and CRC was higher than that in normal tissues (Figure 1).

### 3.2. Concentrations of Serum GOLPH3, CEA, and CA19-9 in GC and CRC Patients and Healthy Controls

**Figure 2:**
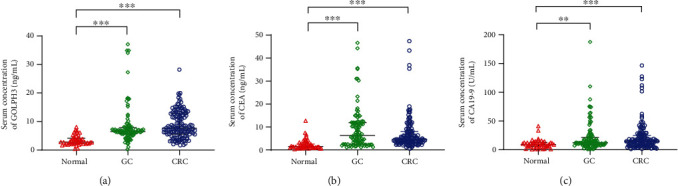
Comparisons of serum concentrations between two groups were performed by the Mann-Whiney *U* test. Comparison of patients and healthy controls, ^∗∗^*P* < 0.01, ^∗∗∗^*P* < 0.001.

The serum GOLPH3 concentration was 6.49 (5.61-7.90) ng/mL in GC patients and 7.67 (5.51-12.75) ng/mL in CRC patients, significantly higher than the 2.78 (2.25-4.12) ng/mL in healthy controls (*P* < 0.001) (Table 1 and Figure 2). Serum CEA and CA19-9 concentrations were also significantly higher in GC and CRC patients than in healthy controls (*P* < 0.01).

### 3.3. Concentration Differences of Serum GOLPH3, CEA, and CA19-9 Based on the Clinical Characteristics of GC and CRC

Concentrations of GOLPH3 in the sera of GC and CRC patients were related to tumor size, tumor invasion depth, cell differentiation, lymph node metastasis, and distant metastasis. In addition, serum concentrations of GOLPH3 in patients with tumor size more than 5 cm, deeper infiltration, poor differentiation, lymph node metastasis, and distant metastasis were significantly higher than those in patients with tumors of less than 5 cm, shallower infiltration, better differentiation, no lymph node metastasis, and no distant metastasis (*P* < 0.05). In contrast, no significant differences in other factors, such as age and sex, were observed for serum GOLPH3 concentrations (Table 2).

### 3.4. Comparison of Serum GOLPH3 between Patients at Different Stages and Healthy Controls

**Figure 3:**
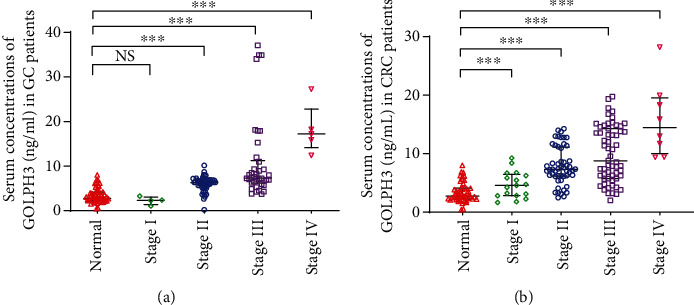
Comparison of serum GOLPH3 levels between patients at different stages and healthy controls. Comparison of patients and healthy controls, ^NS^*P* > 0.05, ^∗∗∗^*P* < 0.001.

Serum GOLPH3 concentrations in GC patients at each individual stage (II-IV) were significantly higher than those in healthy controls (all *P* < 0.001, Figure 3(a)), though there was no significant difference between patients at stage I and healthy controls. Moreover, serum GOLPH3 concentrations in CRC patients at each individual stage (I-IV) were significantly higher than those in healthy controls (all *P* < 0.001, Figure 3(b)), and there was a significant difference in the median serum GOLPH3 concentrations of GC or CRC patients at different stages (*P* < 0.001).

### 3.5. Diagnostic Efficacy of Serum GOLPH3, CEA, and CA19-9 Concentrations in GC and CRC Patients

**Figure 4:**
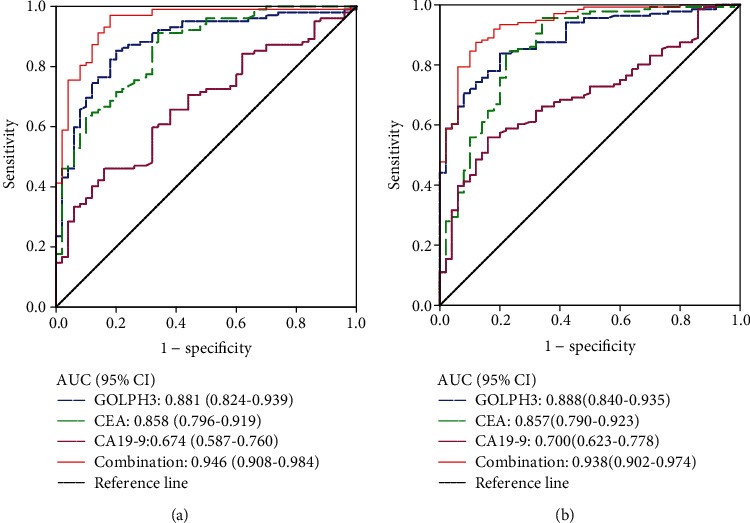
(a) ROC curve of serum GOLPH3, CEA, and CA19-9 concentrations for GC diagnosis. (b) ROC curve of serum GOLPH3, CEA, and CA19-9 concentrations for CRC diagnosis. AUC comparison by DeLong's algorithm indicated that the AUC of the combination of 3 markers was greater than that of each marker.

The AUCs of GOLPH3, CEA, and CA19-9 in GC patients were 0.881 (95% CI: 0.824-0.939, *P* < 0.001), 0.858 (95% CI: 0.796-0.919, *P* < 0.001), and 0.674 (95% CI: 0.587-0.760, *P* < 0.01), respectively. For the ROC curve of GOLPH3 (Figure 4(a)), the Youden index was 0.654, which corresponds to a serum GOLPH3 concentration of 4.24 ng/mL, which is the cutoff value of this study. At this cutoff value (Table 3), the sensitivity of the serum GOLPH3 concentration in GC diagnosis was 85.3%, and the specificity was 80.0%. The AUC of the marker combination was 0.946 (95% CI: 0.908-0.984, *P* < 0.001). Subtraction of AUCs of the combination minus GOLPH3 was 0.065, the *Z* value was 2.693, and *P* < 0.01. The difference in diagnostic value between the combination and GOLPH3 was statistically significant. The diagnostic efficacy of the combination of three markers was higher than that of GOLPH3.

The AUCs of GOLPH3, CEA, and CA19-9 in CRC were 0.888 (95% CI: 0.840-0.935, *P* < 0.001), 0.857 (95% CI: 0.790-0.923, *P* < 0.001), and 0.700 (95% CI: 0.623-0.778, *P* < 0.001), respectively. For the GOLPH3 ROC curve (Figure 4(b)), the Youden index was 0.638, corresponding to the cutoff value of the serum GOLPH3 concentration of 4.24 ng/mL. At this cutoff value (Table 3), the sensitivity of the serum GOLPH3 concentration in the diagnosis of CRC was 83.8%, the specificity was 80.0%, and the marker combination AUC was 0.938 (95% CI: 0.902-0.974, *P* < 0.001). Subtraction of AUCs of the combination minus GOLPH3 was 0.050, the *Z* value was 2.422, and *P* < 0.05. Moreover, the difference in diagnostic value between the combination and GOLPH3 was statistically significant, and the diagnostic efficacy of the combination was higher than that of GOLPH3.

### 3.6. Comparisons of Levels of Serum GOLPH3 in GC and CRC Patients before and after Surgery

Ninety-seven of 102 patients with GC and 128 of 136 patients with CRC underwent radical surgery. Serum samples were collected 1 week (7 ± 2 days) and 1 month (30 ± 2 days) after surgery. The Wilcoxon test was used to analyze the effects of serum GOLPH3 concentrations before and after radical surgery, and the results showed that the difference in serum GOLPH3 concentration before and after radical surgery was statistically significant (*P* < 0.001).

**Figure 5:**
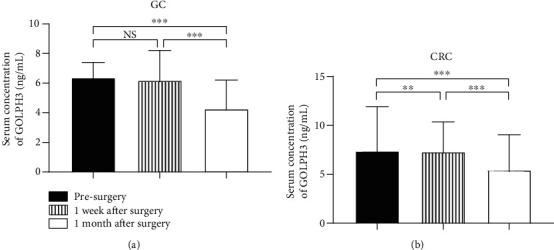
Serum GOLPH3 concentrations were detected in GC and CRC patients before and after radical surgery. The Wilcoxon test was used to analyze the difference of serum GOLPH3 concentration. (a) Concentrations of serum GOLPH3 in GC patients at 1 month after surgery were significantly lower than those before and at 1 week after surgery (*P* < 0.001). (b) Concentrations of serum GOLPH3 in CRC patients at 1 week and 1 month after surgery were significantly lower than those before surgery (*P* < 0.001).

Paired comparisons indicated no statistically significant difference in serum GOLPH3 concentration at 1 week after surgery (median 6.19 ng/mL) and before surgery (median 6.38 ng/mL) in GC patients (*P* = 0.944). Compared with before or 1 week after surgery, the difference was statistically significant at one month after surgery (median 4.27 ng/mL) (*P* < 0.001) (Figure 5(a)). In CRC patients, the difference in serum GOLPH3 concentration at 1 week after (median 7.31 ng/mL) and before (median 7.38 ng/mL) surgery was also significant (*P* < 0.01), as was the difference between before or 1 week after surgery and one month after surgery (median 5.43 ng/mL) (*P* < 0.001) (Figure 5(b)). The *P* value was corrected by the Bonferroni method.

### 3.7. Correlation among Serum GOLPH3, CEA, and CA19-9 Concentrations in GC and CRC Patients

There were no significant correlations between serum GOLPH3 concentrations and serum CEA and CA19-9 concentrations in GC patients. However, serum GOLPH3 concentrations in CRC patients correlated positively with serum CEA and CA19-9 concentrations, and the correlation was statistically significant (Table 4).

## 4. Discussion

Early clinical examination methods for GC and CRC include gastrointestinal endoscopy, gastrointestinal angiography, and tumor markers. Due to its high accuracy, endoscopy is the gold standard for definitive diagnosis of GC and CRC; despite its safety and convenience, it is difficult to use for large-scale screening. In contrast, tumor marker detection has the advantages of being noninvasive, convenient, highly repeatable, and more acceptable to patients. In fact, it is widely used for screening and early diagnosis of gastrointestinal tumors and to help evaluate prognosis.

A large number of international studies have confirmed that GOLPH3 is a protooncogene that is highly expressed in various solid cancer tissues and is related to high malignancy and poor prognosis [6–10]. Many studies have correlated GOLPH3 oncogenicity with the AKT/mTOR, MAPK/ERK, JAK2/STAT3, and Wnt/*β*-catenin signaling pathways. Tumor cell in vitro experiments or experiments in nude mice have clarified the role of the GOLPH3 protein in cancer cell proliferation, metastasis, and angiogenesis [11]. In addition, our previous studies confirmed that GOLPH3 gene expression is upregulated in CRC tissues. GOLPH3 can promote tumor cell proliferation in colon cancer through PI3K/Akt/mTOR and Wnt/*β*-catenin signaling, and its overexpression can be considered an important sign to evaluate the prognosis of CRC [12, 13]. Through a series of experiments, we found that the expression of GOLPH3 was increased in colon cancer HT29 cells resistant to 5-fluorouracil (5-FU). Silencing the GOLPH3 gene enhances the sensitivity of HT29 cells to 5-FU chemotherapy [14], and GOLPH3-induced chemotherapy resistance of HT29 cells to cisplatin involves the same molecular pathway [15].

In this study, we investigated whether GOLPH3 can be used as a tumor marker for early diagnosis and prognosis in patients with GC and CRC. Serum GOLPH3 concentrations in patients with GC and CRC were significantly higher than those in healthy controls. Associations between serum GOLPH3 and clinicopathological parameters were also analyzed. Overall, our findings indicate that serum GOLPH3 may reflect the depth of tumor invasion and correlate with lymph node and distant metastases. Therefore, GOLPH3 may be a good indicator for assessing local lymphatic or distant metastasis of GC and CRC. The difference in serum GOLPH3 concentrations between GC and CRC patients before and after surgery is obvious, indicating that GOLPH3 can be used as a prognostic indicator for GC and CRC.

The tumor markers CEA and CA19-9 are widely utilized in the diagnosis, monitoring, follow-up, and prognosis determination of GC and CRC patients. In general, combined methods of detection can improve prognostic accuracy. For example, preoperative serum CEA, CA19-9, and CA125 levels can be used to predict the resectability of cholangiocarcinoma [16]. Wang et al. found that combined detection of CA125 and NSE may improve diagnostic efficiency for liver metastasis of lung cancer [17]. According to Qiao et al., scCD147 and scMMP-9 could be independent factors for the prediction of chemotherapy outcome, presenting an increase in diagnostic efficiency when combined with scCD147 and scMMP-9 [18]. In our study, the results of ROC curve analysis indicated that serum GOLPH3 has a high AUC. GOLPH3 may thus be applied as a novel tumor marker for GC and CRC and has a higher diagnostic efficacy when combined with CEA and CA19-9 detection.

We also found that in CRC patients, preoperative serum GOLPH3 concentrations correlated positively with the serum CEA and CA19-9 concentrations. The Golgi apparatus is the main site for protein glycosylation, and the sugar chain structure of glycoproteins is essential for protein secretion. They can affect protein folding and mediate transport and protein positioning throughout the secretory pathway [19]. The role of GOLPH3 in tumorigenesis may be related to its involvement in protein transport from the Golgi apparatus to the plasma membrane and increased glycosylation of cancer-associated glycoproteins [3]. We speculate that GOLPH3 may be involved in glycosylation processing and transport of CEA and CA19-9.

In summary, serum GOLPH3 concentrations in patients with GC and CRC were higher than those in healthy controls. Moreover, patients with poor tumor differentiation and late staging had higher serum GOLPH3 concentrations. Combined detection of GOLPH3, CEA, and CA19-9 may be used as a new diagnostic method and an index to evaluate the surgical effect after radical surgery, and GOLPH3 may be used as a novel tumor marker for GC and CRC.

It should be noted that the patients in this study were from a single center and that this study had an insufficient sample size and lacked long-term follow-up. Therefore, further study of GOLPH3 as a prognostic indicator for GC and CRC is needed, and we believe that the correlation between GOLPH3 and gastrointestinal tumors will become clearer in subsequent studies. GOLPH3 may have important value for monitoring the recurrence of GC and CRC and for targeted therapy.

## 5. Conclusions

Serum GOLPH3 concentrations in GC and CRC patients are related to TNM stage. GOLPH3 may represent a novel biomarker for the diagnosis of GC and CRC. The combination of serum GOLPH3, CEA, and CA19-9 concentrations can improve the diagnostic efficiency for GC and CRC. GOLPH3 is expected to become an indicator for the early diagnosis and evaluation of surgical outcome.

## Figures and Tables

**Table 1:** Concentrations of serum GOLPH3, CEA, and CA19-9 in GC and CRC patients and healthy controls.

	GOLPH3 (ng/mL)	CEA (ng/mL)	CA19-9 (U/mL)
Healthy controls	2.78 (2.25-4.12)	1.48 (1.02-2.58)	9.06 (5.56-12.51)
CRC	7.67 (5.51-12.75)^∗∗∗^	4.62 (3.35-8.06)^∗∗∗^	14.47 (7.56-24.86)^∗∗∗^
GC	6.49 (5.61-7.90)^∗∗∗^	6.31 (2.54-11.90)^∗∗∗^	11.86 (8.05-21.48)^∗∗^

^∗∗^ *P* < 0.01, ^∗∗∗^*P* < 0.001.

**Table 2:** Differences in serum GOLPH3 concentration based on clinical characteristics of GC and CRC.

	Gastric cancer			Colorectal cancer		
	No.	Median (IQR25-75)	*P* value	No.	Median (IQR25-75)	*P* value
Sex			0.729			0.120
Male	56	6.34 (5.59-7.98)		66	6.81 (4.33-12.87)	
Female	46	6.80 (5.90-7.67)		70	7.95 (6.26-12.75)	
Age			0.232			0.058
<60	36	6.33 (5.59-7.17)		46	6.40 (4.36-11.87)	
≥60	66	6.78 (5.57-8.13)		90	7.82 (6.25-13.12)	
Tumor size			0.000			0.000
<5 cm	70	6.32 (5.49-6.90)		58	6.80 (4.36-8.63)	
≥5 cm	32	7.98 (6.54-14.54)		78	9.07 (6.25-13.77)	
Differentiation			0.001			0.000
G1-G2	78	6.35 (5.49-7.14)		110	7.09 (4.58-11.24)	
G3	24	8.63 (6.19-16.86)		26	13.54 (7.84-14.93)	
T stage			0.017			0.001
T1-T2	29	6.35 (3.67-6.89)		27	5.86 (3.25-8.39)	
T3-T4	73	6.69 (5.81-8.79)		109	7.96 (6.23-13.06)	
N stage			0.000			0.000
Negative	16	5.12 (2.58-6.20)		71	7.04 (4.59-8.75)	
Positive	86	6.81 (6.06-8.21)		65	9.56 (6.25-14.78)	
M stage			0.001			0.001
M0	97	6.38 (5.42-7.41)		128	7.38 (5.45-11.93)	
M1	5	17.18 (14.13-22.75)		8	14.47 (10.02-19.55)	

**Table 3:** Diagnostic efficacy of GOLPH3, CEA, and CA19-9 for GC and CRC.

Tumor markers	Sensitivity (%)	Specificity (%)	AUC	SE	95% CI	*P* value
Gastric cancer						
GOLPH3	85.3	80.0	0.881	0.029	0.824-0.939	<0.001
CEA	57.8	92.0	0.858	0.031	0.796-0.919	<0.001
CA19-9	14.7	98.0	0.674	0.044	0.587-0.760	<0.01
Combination	97.1	82.0	0.946	0.019	0.908-0.984	<0.001
Colorectal cancer						
GOLPH3	83.8	80.0	0.888	0.024	0.840-0.935	<0.001
CEA	44.4	92.0	0.857	0.034	0.790-0.923	<0.001
CA19-9	14.7	98.0	0.700	0.040	0.623-0.778	<0.001
Combination	87.5	88.0	0.938	0.019	0.902-0.974	<0.001

**Table 4:** Correlation between three different tumor markers in GC and CRC.

	GOLPH3 vs. CEA		GOLPH3 vs. CA19-9		CEA vs. CA19-9	
	*ρ* ^∗^	*P* value	*ρ*	*P* value	*ρ*	*P* value
Gastric cancer	0.189	0.057	0.078	0.437	0.108	0.279
Colorectal cancer	0.196	0.022	0.313	<0.001	0.307	<0.001

^∗^Spearman's correlation coefficient.

## Data Availability

The data used to support the findings of this study are available from the corresponding author upon request.
